# A US-based national surveillance study for the susceptibility and epidemiology of *Clostridioides difficile* isolates with special reference to ridinilazole: 2020–2021

**DOI:** 10.1128/aac.00349-23

**Published:** 2023-09-20

**Authors:** D. R. Snydman, L. A. McDermott, C. M. Thorpe, E. J. C. Goldstein, A. N. Schuetz, S. Johnson, D. N. Gerding, L. Gluck, D. Bourdas, K. C. Carroll, C. K. Lancaster, K. W. Garey, Q. Wang, S. T. Walk, E. Duperchy

**Affiliations:** 1 Tufts Medical Center, Boston, Massachusetts, USA; 2 Tufts University School of Medicine and the Stuart B. Levy Center for the Integrated Management of Antimicrobial Resistance, Boston, Massachusetts, USA; 3 Kindred Health Care System, Rancho Cucamonga, California, USA; 4 Mayo Clinic School of Medicine and Science, Rochester, Minnesota, USA; 5 Edward Hines, Jr. VA Hospital, Hines, Illinois, USA; 6 Loyola University Medical Center, Maywood, Illinois, USA; 7 Johns Hopkins University School of Medicine, Baltimore, Maryland, USA; 8 University of Houston College of Pharmacy, Houston, Texas, USA; 9 Summit (Oxford) Ltd, Abingdon, United Kingdom; 10 Montana State University, Bozeman, Montana, USA; 11 Johns Hopkins Hospital, Baltimore, Maryland, USA; Bill & Melinda Gates Medical Research Institute, Cambridge, Massachusetts, USA

**Keywords:** ridinilazole, *in vitro* activity, *Clostridioides difficile*, US surveillance studies, ribotyping

## Abstract

We have previously reported on the susceptibility and epidemiology of *Clostridioides difficile* isolates from six geographically dispersed medical centers in the United States. This current survey was conducted with isolates collected in 2020–2021 from six geographically dispersed medical centers in the United States, with specific attention to susceptibility to ridinilazole as well as nine comparators. *C. difficile* isolates or stools from patients with *C. difficile* antibiotic-associated diarrhea were collected and referred to a central laboratory. After species confirmation of 300 isolates at the central laboratory, antibiotic susceptibilities were determined by the agar dilution method [M11-A9, Clinical and Laboratory Standards Institute (CLSI)] against the 10 agents. Ribotyping was performed by PCR capillary gel electrophoresis on all isolates. Ridinilazole had a minimum inhibitory concentration (MIC) 90 of 0.25 mcg/mL, and no isolate had an MIC greater than 0.5 mcg/mL. In comparison, fidaxomicin had an MIC 90 of 0.5 mcg/mL. The vancomycin MIC 90 was 2 mcg/mL with a 0.7% resistance rate [both CLSI and European Committee on Antimicrobial Susceptibility Testing (EUCAST) criteria]. The metronidazole MIC 90 was 1 mcg/mL, with none resistant by CLSI criteria, and a 0.3% resistance rate by EUCAST criteria. Among the 50 different ribotypes isolated in the survey, the most common ribotype was 014–020 (14.0%) followed by 106 (10.3%), 027 (10%), 002 (8%), and 078–126 (4.3%). Ridinilazole maintained activity against all ribotypes and all strains resistant to any other agent tested. Ridinilazole showed excellent *in vitro* activity against *C. difficile* isolates collected between 2020 and 2021 in the United States, independent of ribotype.

## INTRODUCTION

*Clostridioides* (formerly *Clostridium) difficile* infection (CDI) continues to be a problem worldwide (1 – 3) causing substantial morbidity and mortality. It is currently listed among the top five threats in antimicrobial resistance in the United States listed by the Centers for Disease Control (CDC) (3, 4). In the most recent threat report, CDC estimates that approximately 223,900 annual cases of CDI require hospitalization in the United States, with approximately 12,800 deaths (4). Although these numbers have decreased slightly since 2013 (5), *C. difficile* is still ranked number three nationally among all the antimicrobial resistance threats (5).

The appearance of the hypervirulent fluoroquinolone-resistant NAP1/BI/027 isolates, which have been associated with epidemics of complicated CDI cases, toxic megacolon, and increased mortality, has only further highlighted the urgency of our need to understand the epidemiology of *C. difficile* in the United States (6).

Ridinilazole is under development for the treatment of CDI (7, 8). Ridinilazole is a more-narrow spectrum antibiotic than some antibiotics currently used to treat CDI, namely vancomycin and metronidazole. Clinical studies have also shown that ridinilazole is associated with less disturbance to the gut microbiota in patients with CDI compared to the standard of care, vancomycin (9). Vancomycin has a profound impact on the microbiota in the gut causing prolonged and more significant dysbiosis and loss of secondary bile acids, features known to be associated with higher relapse rates (9, 10). Furthermore, based on one small study, ridinilazole may preserve the gut microbiota better than fidaxomicin, both of which have less impact on gut microbiota than vancomycin (9, 11, 12).

We have previously been involved with national surveillance of *C. difficile* susceptibility to various panels of agents, along with REA typing and toxin gene profiling since 2011 (13, 14). As part of a surveillance program in 2020–2021, we undertook a 300-isolate study of *C. difficile* from six different geographically dispersed medical centers in the United States. *In vitro,* antimicrobial susceptibility test of these 300 contemporary *C. difficile* isolates to ridinilazole and comparators and their molecular typing using PCR ribotyping was performed.

## RESULTS

Ridinilazole had an excellent activity against the 300 isolates tested (Table 1). The MIC 90 was 0.25 mcg/mL. In comparison, fidaxomicin had an MIC 90 of 0.5 mcg/mL. Vancomycin had an MIC 90 of 2 mcg/mL. Vancomycin resistance was seen with 0.7% of isolates based on both M11-A9 Clinical and Laboratory Standards Institute (CLSI) and European Committee on Antimicrobial Susceptibility Testing (EUCAST) criteria. The metronidazole MIC 90 was 1 mcg/mL with none resistant by CLSI criteria, and with very little resistance noted (0.3%) by EUCAST criteria. For other notable antibiotics, significant resistance was seen. For clindamycin, the MIC 90 was greater than 32 mcg/mL with 26% resistance according to CLSI breakpoints. The moxifloxacin MIC 90 was 16 mcg/mL with 14.7% resistance noted. Imipenem had an MIC 90 of 8 mcg/mL and accompanying resistance of 5%. Rifampin and rifaximin had very low MIC 90’s (Table 1). Tigecycline had an MIC 90 of 0.12 mcg/mL with an ECOFF resistance rate of 0.7%.

**TABLE 1:** Activities of the antimicrobial agents against 300 *C. difficile* isolates

Antimicrobial agent	MIC range (mcg/mL)	MIC 50 (mcg/mL)	MIC 90 (mcg/mL)	Percent CLSI	Resistant EUCAST
Ridinilazole	0.03–0.5	0.25	0.25	NA^*a*^	NA^*a*^
Fidaxomicin	0.03–0.5	0.25	0.5	NA^*a*^	NA^*a*^
Rifaximin	<0.004– >4	0.015	0.03	NA^*a*^	NA^*a*^
Rifampin	<0.004– >4	<0.004	0.008	NA^*a*^	15.0%^*b*^
Tigecycline	<0.06–0.5	0.12	0.12	0.0%^*c*^	0.7%^*b*^
Vancomycin	0.25–4	2	2	0.7%^*b*^	0.7%^*b*^
lmipenem	2–16	4	8	5.0%	NA^*a*^
Moxifloxacin	1–32	2	16	14.7%	14.7%^*b*^
Metronidazole	0.12–4	0.5	1	0.0%	0.3%^*b*^
Clindamycin	0.5– >32	4	>32	26.0%	NA^*a*^

^*a*^ NA, not applicable. CLSI or EUCAST recommended breakpoint for resistance not available.

^*b*^ TheCLSI or EUCAST, as applicable, ECOFF value was applied, in the absence of a clinical breakpoint.

^*c*^ For tigecycline, the breakpoint for resistance recommended for anaerobes by the FDA was used.

**Fig 1:**
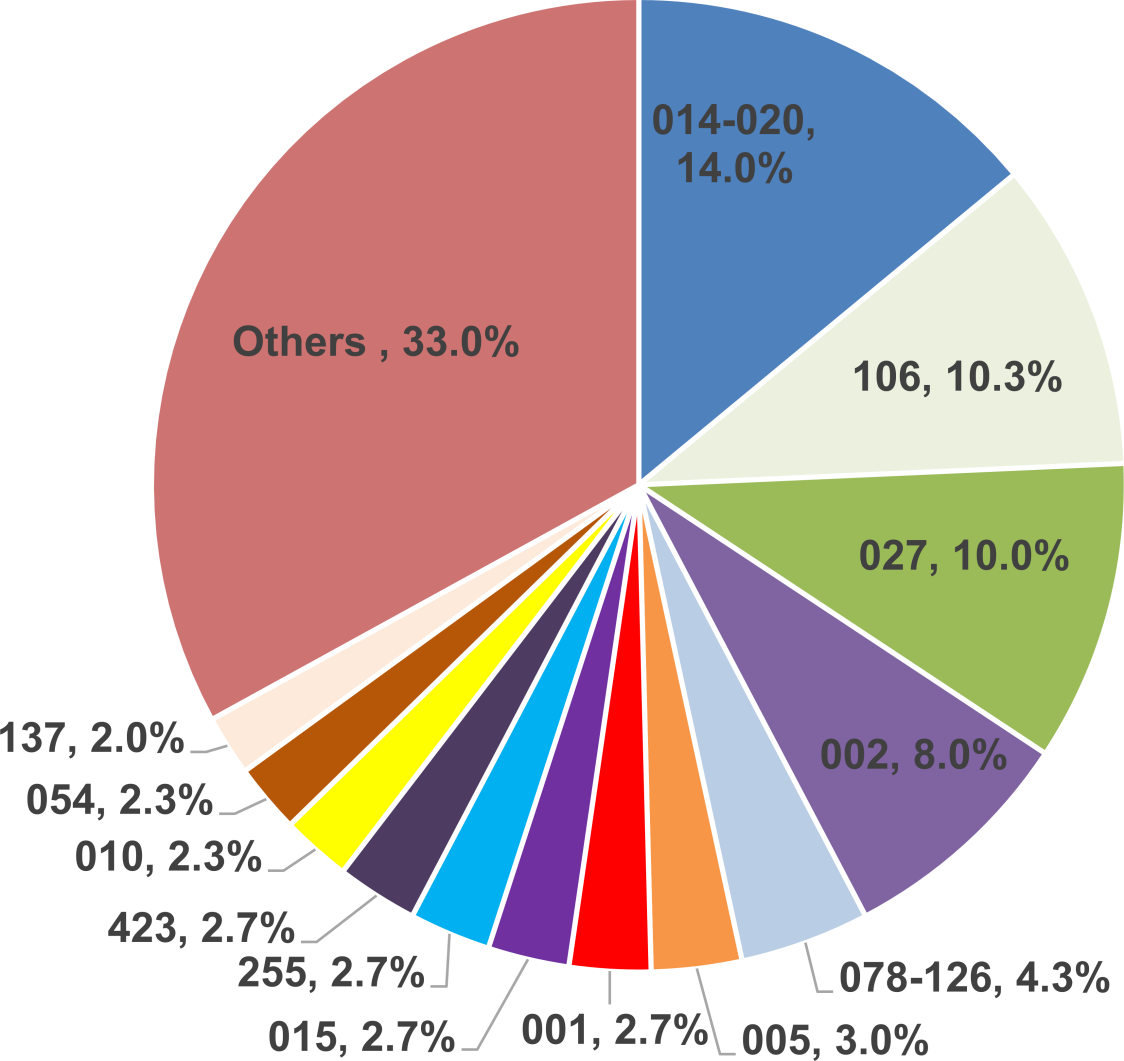
Distribution of ribotypes in the survey of 2020–2021.

The distribution of isolated ribotypes is shown in Fig. 1. There were over 50 different ribotypes seen in the survey. The most common ribotype was 014–020 (14.0%) followed by 106 (10.3%), 027 (10.0%), 002 (8.0%), and 078–126 (4.3%). We did find that 2.3% of the isolates were a non-toxigenic ribotype, namely 010 (15). Presumably, these isolates were chosen from the stool of a patient with a mixed *C. difficile* population, which occurs in approximately 16% of cases (16).

When one examines the ribotype distribution in comparison to 2016, we saw a change in distribution with an increase in 014–020 from 11.8% to 14.0%, whereas we saw a 50% decrease in ribotype 106 (from 15.0% to 10.3 %) and a 30% decrease in ribotype 027 (from 13.1% to 10.0%) (17).

**Fig 2:**
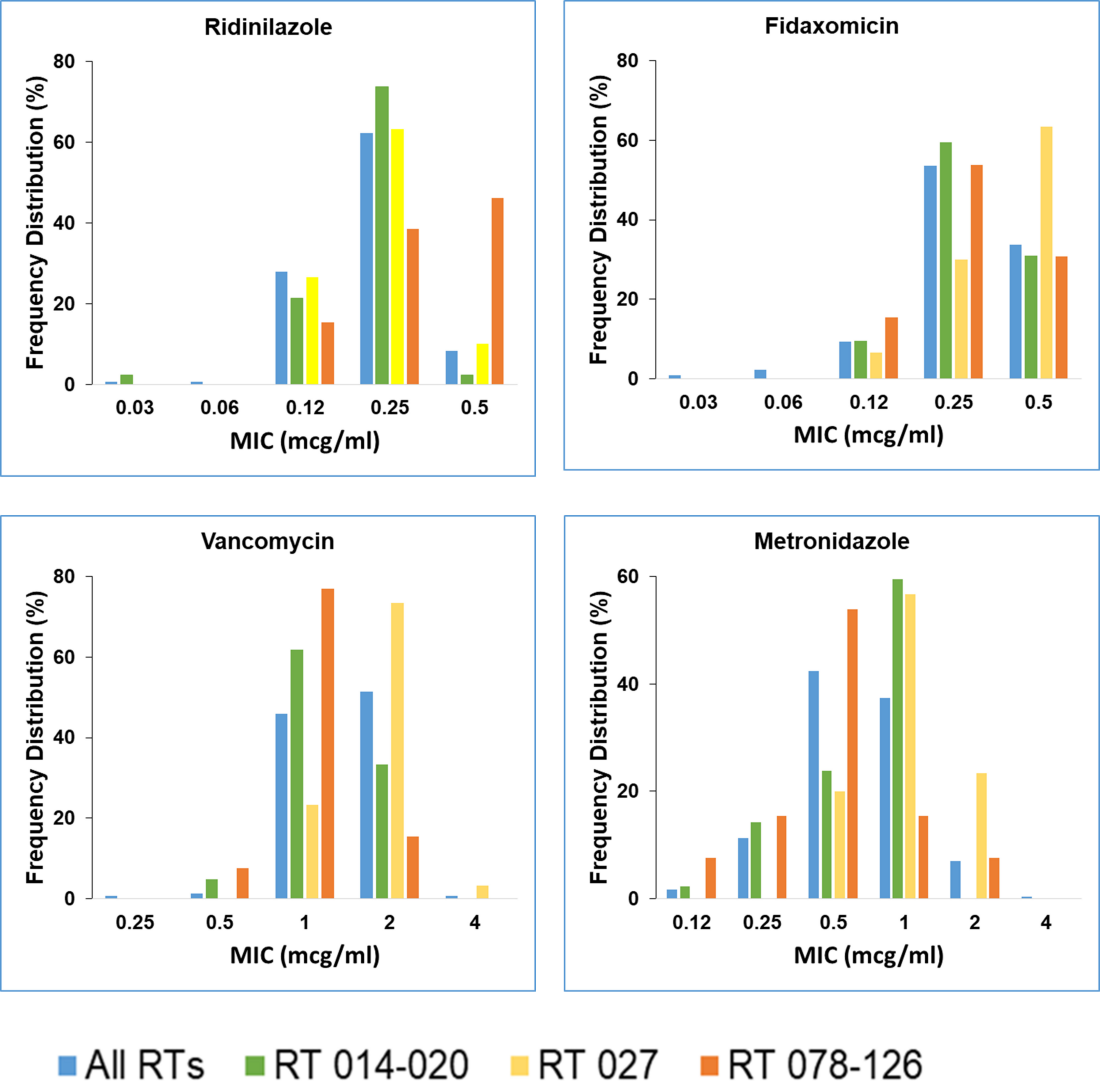
Distribution of MIC values for ridinilazole, fidaxomicin, vancomycin and metronidazole against all ribotypes: the non-hypervirulent ribotype 014–20 and the hypervirulent ribotypes 027 and 078–126.

The activity of ridinilazole was maintained across all ribotypes including the hypervirulent ribotypes 027 and 078–126 (Table 2). The distribution of MIC values is shown in Fig. 2 for ridinilazole, fidaxomicin, vancomycin, and metronidazole. Only 25 isolates (8.3%) showed a ridinilazole MIC value of 0.5 mcg/mL, that is, higher than ridinilazole MIC 90, which included nine hypervirulent ribotypes (6 RT 078–126 and 3 RT 027). There were two isolates resistant to vancomycin with an MIC of 4 mcg/mL, one was ribotype 027 and the other was ribotype 053–163.

**TABLE 2:** Activity of the antimicrobial agents by ribotype for groups with more than 10 isolates (mcg/mL)

Ribotype		RDZ^*a*^	FDX	RFX	RIF	TGC	VAN	IMI	MOX	MTZ	CC
	Range tested→ (mcg/mL)	4–0.004	4–0.004	4–0.004	4–0.004	4–0.06	32–0.25	16–0.12	32–0.5	16–0.06	32–0.5
014–020, *N* = 42^*b*^	Range	0.03–0.5	0.12–0.5	<0.004–0.03	<0.004–0.008	<0.06–0.12	0.5–2	2–8	1–8	0.12–1	2–8
	MIC 50	0.25	0.25	0.015	<0.004	0.12	1	4	2	1	4
	MIC 90	0.25	0.5	0.03	0.008	0.12	2	8	2	1	4
	%R CLSI	NA	NA	NA	NA	0.0%	0.0%	0.0%	4.8%	0.0%	9.5%
	%R EUCAST	NA	NA	NA	11.9%	0.0%	0.0%	NA	4.8%	0.0%	NA
078–126, *N* = 13	Range	0.12–0.5	0.12–0.5	<0.004–0.03	<0.004–0.008	<0.06–0.12	0.5–2	2–8	1–16	0.12–2	2–>32
	MIC 50	0.25	0.25	0.015	<0.004	0.12	1	4	2	0.5	4
	MIC 90	0.5	0.5	0.03	<0.004	0.12	2	8	2	1	8
	%R CLSI	NA	NA	NA	NA	0.0%	0.0%	0.0%	7.7%	0.0%	38.5%
	%R EUCAST	NA	NA	NA	7.7%	0.0%	0.0%	NA	7.7%	0.0%	NA
027, *N* = 30	Range	0.12–0.5	0.12–0.5	<0.004– >4	<0.004– >4	<0.06–0.12	1–4	4–16	1–32	0.5–2	2–>32
	MIC 50	0.25	0.5	0.03	<0.004	<0.06	2	8	16	1	>32
	MIC 90	0.25	0.5	>4	>4	0.12	2	16	32	2	>32
	%R CLSI	NA	NA	NA	NA	0.0%	3.3%	20.0%	63.3%	0.0%	80.0%
	%R EUCAST	NA	NA	NA	40.0%	0.0%	3.3%	NA	63.3%	0.0%	NA
106, *N* = 31	Range	0.12–0.5	0.12–0.5	0.008–0.03	<0.004–0.008	<0.06–0.25	1–2	2–16	1–32	0.25–2	1–8
	MIC 50	0.25	0.5	0.015	<0.004	0.12	1	8	2	1	4
	MIC 90	0.25	0.5	0.03	0.008	0.12	2	8	2	1	8
	%R CLSI	NA	NA	NA	NA	0.0%	0.0%	9.7%	6.5%	0.0%	16.1%
	%R EUCAST	NA	NA	NA	22.6%	0.0%	0.0%	NA	6.5%	0.0%	NA
002, *N* = 24	Range	0.25–0.5	0.25–0.5	0.015 to >4	<0.004 to >4	<0.06–0.12	0.25–2	2–16	1–32	0.25–2	<0.5 to >32
	MIC 50	0.12	0.25	0.015	<0.004	<0.06	2	4	2	0.5	4
	MIC 90	0.25	0.5	0.03	0.008	0.12	2	8	2	1	8
	%R CLSI	NA	NA	NA	NA	0.0%	0.0%	4.2%	8.3%	0.0%	12.5%
	%R EUCAST	NA	NA	NA	16.7%	0.0%	0.0%	NA	8.3%	0.0%	NA

^*a*^ RDZ: ridinilazole; FDX, fidaxomicin; RFX, rifaximin; RIF, rifampin; TGC tigecycline; VAN, vancomycin; IMI, imipenem; MOX, moxifloxacin; MTZ, metronidazole; CC, clindamycin.

^*b*^ N: number of isolates with ribotype.

Examination of activity against isolates resistant to any of the agents tested demonstrated that ridinilazole maintained its activity (Table 3). Ridinilazole retained activity against isolates resistant to moxifloxacin, clindamycin, imipenem, tigecycline, and vancomycin based on CLSI breakpoints or ECOFF values for EUCAST.

**TABLE 3:** Activity of the antimicrobial agents against isolates demonstrating antimicrobial resistance

Isolates with resistance to:		RDZ^*a*^	FDX	RFX	RIF	TGC	VAN	IMI	MOX	MTZ	CC
Moxifloxacin, *N* = 44^*b*^	MIC 50	0.25	0.25	0.03	<0.004	<0.06	2	8	16	1	>32
	MIC 90	0.25	0.5	>4	>4	0.12	2	16	32	2	>32
	%R CLSI	NA	NA	NA	NA	0.0%	4.5%	22.7%	100.0%	0.0%	75.0%
	%R EUCAST	NA	NA	NA	45.5%	4.5%	4.5%	NA	100.0%	2.3%	NA
Imipenem, *N* = 15	MIC 50	0.25	0.25	0.015	<0.004	0.06	2	16	16	1	64
	MIC 90	0.25	0.5	>4	>4	0.12	2	16	32	2	64
	%R CLSI	NA	NA	NA	NA	0.0%	6.7%	100.0%	66.7%	0.0%	73.3%
	%R EUCAST	NA	NA	NA	33.3%	0.0%	6.7%	NA	66.7%	0.0%	NA
Clindamycin, *N* = 78	MIC 50	0.25	0.25	0.015	<0.004	0.12	2	8	2	1	>32
	MIC 90	0.5	0.5	>4	>4	0.12	2	16	32	2	>32
	%R CLSI	NA	NA	NA	NA	0.0%	2.6	14.1%	42.3%	0.0%	100.0%
	%R EUCAST	NA	NA	NA	29.5%	1.3%	2.6%	NA	42.3%	1.3%	NA
	MIC 50	0.25	0.5	0.03	0.008	0.06	2	8	2	1	8
Rifampin, *N* = 45Tigecycline, N = 2	MIC 90	0.25	0.5	8	8	0.12	2	16	32	2	64
	%R CLSI	NA	NA	NA	NA	0.0%	2.2%	0.0%	44.4%	0.0%	51.1%
	%R EUCAST	NA	NA	NA	100.0%	0.0%	2.2%	NA	44.4%	2.2%	NA
	MICs	0.12, 0.25	0.06, 0.25	0.015, 0.12	Both <0.004	Both 0.5	Both 2	4, 8	Both 32	0.5, 2	2, >64
Vancomycin *N* = 2	%R CLSI	NA	NA	NA	NA	0.0%	0.0%	0.0%	100%	0.0%	50.0%
	%R EUCAST	NA	NA	NA	0.0%	100%	0.0%	NA	100%	0.0%	NA
	MICs	0.12, 0.25	Both 0.12	<0.004, >4	<0.004, >4	Both <0.06	Both 4	8, 16	16, 32	0.5, 1	Both >32
	%R CLSI	NA	NA	NA	NA	0.0%	100%	50.0%	100%	0.0%	100.0%
	%R EUCAST	NA	NA	NA	50.0%	0.0%	100.0%	NA	100%	0.0%	NA

^*a*^ RDZ: ridinilazole; FDX, fidaxomicin; RFX, rifaximin; RIF, rifampin; TGC tigecycline; VAN, vancomycin; IMI, imipenem; MOX, moxifloxacin; MTZ, metronidazole; CC, clindamycin.

^*b*^ N: number of isolates with ribotype.

## DISCUSSION

Ridinilazole showed an excellent *in vitro* activity against contemporary *C. difficile* isolates obtained between 2020 and 2021 from six geographically dispersed medical centers in the United States. No isolate had an MIC greater than 0.5 mcg/mL. Ridinilazole showed more *in vitro* potency than vancomycin or fidaxomicin, currently the preferred agent in the treatment of CDI (18). Clinical development of ridinilazole is on hold due to the Phase 3 Ri-CoDIFy study results. In this study, ridinilazole treatment achieved a higher sustained clinical response compared to vancomycin; however, the study did not pass the statistical threshold for superiority and, as such, the primary endpoint was not achieved (19).

Furthermore, we demonstrated that ridinilazole maintained activity against hypervirulent ribotypes as well as those that were resistant to one or more antimicrobial agents, either used to treat *C. difficile-*associated diarrhea or those used to treat anaerobic infections. Among these hypervirulent strains, many isolates clustered around the vancomycin EUCAST breakpoint of 2 mcg/mL. Most notably, Ribotype 078–126 appears to have the largest dispersion of MICs compared to the other ribotypes. RT078 represents a more diverse group than most other ribotypes, like RT027. These isolates are commonly observed in the environment and agricultural animals. Our results may reflect a trend toward increased resistance or it may also reflect a higher probability of resistance simply due to increased diversity (20 – 22).

When one looks at the trends in ribotype distribution, compared to 2016, there has been a change in ribotype distribution in the United States with a decline in the hypervirulent strains. This has been noted as well by others (23).

In conclusion based on the activity profile, ridinilazole has maintained its activity against *C. difficile* over the past few years of development and is a potential additional agent in the armamentarium to treat *C. difficile-*associated diarrhea.

## MATERIALS AND METHODS

### Medical centers

Stools from patients diagnosed with CDI, or isolates recovered from stools of patients diagnosed with CDI, were referred to the Special Studies Laboratory at Tufts Medical Center by six medical centers for strain isolation and speciation (stools) or speciation (isolates) and subsequent susceptibility testing (Table 4). The medical centers were as follows: Hines VA Hospital, Chicago, IL; Mayo Clinic, Rochester, MN; Johns Hopkins University School of Medicine, Baltimore MD; Tufts Medical Center, Boston, MA; Kindred Health System, Rancho Cucamonga, CA; and the University of Houston College of Pharmacy, Houston, TX. Each center has both excellent anaerobic bacteriology laboratories and investigators willing to collaborate.

**TABLE 4:** Participating study centers

Medical center	State	Principal investigator	Type of sample	Number of isolates
Hines VA Hospital	Illinois	S. Johnson, MD	Isolate	52
Johns Hopkins	Maryland	Karen C. Carroll, MD	Isolate	46
Kindred Health System	California	Ellie JC. Goldstein, MD	Stool	25
Mayo Clinic	Minnesota	Audrey N. Schuetz, MD	Stool	49
Tufts Medical Center	Massachusetts	David R. Snydman, MD	Isolate	81
University of Houston College of Pharmacy	Texas	Kevin W. Garey, PharmD, MS	Isolate	47

### Bacterial isolates

Convenience samples from patients with stools that tested positive for *C. difficile* toxin were obtained from Johns Hopkins, Tufts Medical Center, the University of Houston College of Pharmacy, and Hines VA hospital. These centers performed *C. difficile* stool culture following their standard procedures to obtain isolates and maintained the isolates stocked at −80°C. Isolates were cultured into pre-reduced anaerobically sterilized chopped meat broth (Anaerobe Systems, Morgan Hill, CA, USA) or other appropriate media for transport prior to shipment. Shipment of isolates was at ambient temperature. The isolates were kept at room temperature in the transport media as received by Tufts Medical Center until tested.

Two centers, namely Mayo Clinic (Minnesota) and Kindred Healthcare (California), provided frozen stool samples from patients who had *C. difficile* toxin-positive samples. De-identified toxin-positive stool samples were stored at −80°C on site until being shipped overnight on dry ice to the Special Studies Laboratory at Tufts Medical Center. Upon receipt by Tufts the samples were stored at −80°C until processing.

Each institution that performed isolation of *C. difficile* was instructed to send an average of 50 isolates collected throughout the study period, July 2020–August 2021 while those providing stool were asked to send approximately 55 samples. Due to resource allocation during the COVID-19 pandemic, not all centers were able to provide the requisite number of samples. Additional isolates from Tufts Medical Center obtained at the appropriate time interval were included to complete a total of 300 isolates to be tested.

### Processing of samples and identification of isolates

At the central laboratory, both the confirmation of the isolate as *C. difficile* and the culture of stool were accomplished by plating on *C. difficile* selective medium (cycloserine–cefoxitin–fructose agar with taurocholate; Anaerobe Systems, Morgan Hill, CA, USA) and observing for characteristic colonial morphology (24, 25). Thawed stool samples were ethanol shocked prior to being plated (25). A proline disc test (Remel Products, Lenexa, KS, USA) and gram stain were performed. This was followed by using the identifying method API20A^®^ (BioMerieux Inc., Durham, NC, USA).

Speciated isolates were stored by using a cell paste swabbed from fresh plates that were suspended directly into skim milk and frozen at −80°C for future testing and reference (26).

### Antimicrobial susceptibility testing

The MICs of the isolates were determined in singlicate using the CLSI-recommended agar dilution methodology against the panel of antibiotics shown in Table 5 (26, 27). The medium was brucella agar (BD BBL, Franklin Lakes, NJ, USA) supplemented with 5 mg hemin, 1 mg Vitamin K_1_ per liter, and 5% (vol/vol) lysed sheep blood. The antibiotic-containing plates were prepared freshly on the morning of the test. The inocula were prepared using direct suspension to achieve turbidity equivalent to a 0.5 McFarland standard and distributed accordingly into a Steers replicating block that deposited the inocula onto the test plates. The inocula density on the agar surface was ~10^4^ CFU/spot. The plates were incubated for 44–48 hours at 35°C−37°C in an anaerobic chamber with an atmosphere of 85% N_2_, 5% CO_2_, and 10% H_2_. After incubation, the plates were examined against a dark, non-reflecting background and the MIC endpoints read at the concentration where a marked reduction occurred in the appearance of growth on the test plate as compared to that of growth on the control plate. Tests were to be repeated if the MICs of the control organisms were outside of the CLSI-specified range (27). The control and reference organisms used in this study were *C. difficile* ATCC 700057, *C. difficile* ATCC 43255, *Bacteroides thetaiotaomicron* ATCC 29741, *Bacteroides fragilis* ATCC 25285, and *Staphylococcus aureus* ATCC 29213. Isolates with high MICs to ridinilazole (≥0.5 mcg/mL), fidaxomicin (≥2 mcg/mL), vancomycin (≥4 mcg/mL), metronidazole (≥4 mcg/mL), or tigecycline (≥1 mcg/mL) were retested twice on separate days of testing. If the results were not identical, the isolate was tested a third time. There were no isolates with high MIC’ to fidaxomicin or ridinilazole that met these criteria for re-testing.

**TABLE 5:** Antimicrobial agents tested, ranges, and breakpoints for susceptibility

Antimicrobial agent	Abbreviation	Range tested (mcg/mL)	Breakpoint (mcg/mL)	
			CLSI	EUCAST
Ridinilazole	RDZ	4–0.004	NA^*a*^	NA^*a*^
Fidaxomicin	FDX	4–0.004	NA^*a*^	NA^*a*^
Rifaximin	RFX	4–0.004	NA^*a*^	NA^*a*^
Rifampin	RIF	4–0.004	NA^*a*^	>0.004^*b*^
Tigecycline	TGC	4–0.004	>16^*c*^	>0.25^*b*^
Metronidazole	MTZ	16–0.06	>32	>2^*b*^
Vancomycin	VAN	32–0.25	>4^*a*^	>2^*b*^
Imipenem	IMI	16–0.12	>16	NA^*a*^
Moxifloxacin	MOX	32–0.5	>8	>4^*b*^
Clindamycin	CC	32–0.5	>8	NA

^*a*^ NA, not applicable. CLSI or EUCAST recommended breakpoint for resistance not available.

^*b*^ The CLSI or EUCAST, as applicable, ECOFF value was applied, in the absence of a clinical breakpoint.

^*c*^ For tigecycline, the breakpoint for resistance recommended for anaerobes by the FDA was used.

The rates of resistance of the antimicrobial agents were determined using currently accepted CLSI breakpoints for anaerobes (27). For agents that did not have CLSI recommendations, FDA recommendations or the manufacturer’s proposed breakpoint(s) were used (Table 5) (28). We also looked at rates of resistance using EUCAST breakpoints, based on epidemiologic cutoff values (ECOFF), which have been established for *C. difficile* (29).

### Ribotyping

All isolates were sent to the Walk Laboratory, Montana State University, for amplicon preparation and analysis. A template for amplicon generation was obtained by growing *C. difficile* isolates overnight under anaerobic conditions in pre-reduced brain heart infusion broth supplemented with 0.1% cysteine. PCR was conducted directly on 10-fold diluted cultures (in sterile H_2_O). Fluorescently labeled amplicons were generated using PCR directly on 10-fold diluted cultures (in sterile H_2_O) (Promega MasterMix, M7502, Promega, Madison, WI, USA) with forward and reverse primers at 10 pmol/mcl. The forward primer was 5-GTGCGGCTGGATCACCTCCT-3 and reverse primer was 5–6-FAM/CCCTGCACCCTTAATAACTTGACC. Thermocycling conditions included 95°C for 10 min followed by 35 cycles of 95°C for 0.5 min, 55°C for 0.5 min, 72°C for 1.5 min, and a final elongation step of 72°C for 10 min. Amplicons were stored at −20°C until fragment analysis using a Promega Spectrum Compact CE System (Promega). For CE analysis, amplicons were diluted in sterile DNase-/RNase-free water, Hi-DI Formamide (Life Technologies, Rockville, MD, USA) and ROX1000 size standard (Bioventure Inc, Murfreesboro, TN, USA) and added to a CE loading plate. Resulting .fsa files were analyzed using the Walk Lab CdiffFragR pipeline (https://github.com/nvpinkham/CdiffFragR) against the in-house database of ribotyping profiles (version F-Ribotyping_Pes.lite.15), as previously described (16, 17, 30).

## References

[1] Burke KE , Lamont JT . 2014. Clostridium difficile infection: a worldwide disease. Gut Liver 8:1–6. doi:10.5009/gnl.2014.8.1.1 24516694 PMC3916678

[2] Borren NZ , Ghadermarzi S , Hutfless S , Ananthakrishnan AN . 2017. The emergence of Clostridium difficile infection in Asia: a systematic review and meta-analysis of incidence and impact. PLoS ONE 12:e0176797. doi:10.1371/journal.pone.0176797 28463987 PMC5413003

[3] Lessa FC , Winston LG , McDonald LC , Emerging Infections Program C. difficile Surveillance Team . 2015. Burden of Clostridium difficile infection in the United States. N Engl J Med 372:2369–2370. doi:10.1056/NEJMc1505190 26061850 PMC10880113

[4] Centers for Disease Control and Prevention . 2019. Antibiotic resistance threats in the United States. US Department of Health and Human Services, Washington, DC.

[5] Centers for Disease Control and Prevention . 2013. Antibiotic resistance threats in the United States. US Department of Health and Human Services, Washington, DC.

[6] Pépin J , Valiquette L , Cossette B . 2005. Mortality attributable to nosocomial Clostridium difficile-associated disease during an epidemic caused by a hypervirulent strain in Quebec. Can Med Assoc J 173:1037–1042. doi:10.1503/cmaj.050978 16179431 PMC1266326

[7] Goldstein EJC , Citron DM , Tyrrell KL , Merriam CV . 2013. Comparative in vitro activities of SMT19969, a new antimicrobial agent, against Clostridium difficile and 350 gram-positive and gram-negative aerobic and anaerobic intestinal flora isolates. Antimicrob Agents Chemother 57:4872–4876. doi:10.1128/AAC.01136-13 23877700 PMC3811411

[8] Vickers RJ , Tillotson GS , Nathan R , Hazan S , Pullman J , Lucasti C , Deck K , Yacyshyn B , Maliakkal B , Pesant Y , Tejura B , Roblin D , Gerding DN , Wilcox MH , CoDIFy study group . 2017. Efficacy and safety of ridinilazole compared to vancomycin for the treatment of C. difficile imfection: a phase 2, randomized, double-blind, active-controlled, non-inferiority study. Lancet Infect Dis 17:735–744. doi:10.1016/S1473-3099(17)30235-9 28461207 PMC5483507

[9] Thorpe CM , Kane AV , Chang J , Tai A , Vickers RJ , Snydman DR . 2018. Enhanced preservation of the human intestinal microbiota by ridinilazole, a novel Clostridium difficile-targeting antibacterial, compared to vancomycin. PLoS One 13:e0199810. doi:10.1371/journal.pone.0199810 30071046 PMC6071993

[10] Qian X , Yanagi K , Kane AV , Alden N , Lei M , Snydman DR , Vickers RJ , Lee K , Thorpe CM . 2020. Ridinilazole, a narrow spectrum antibiotic for treatment of Clostridioides difficile infection, enhances preservation of microbiota-dependent bile acids. Am J Physiol Gastrointest Liver Physiol 319:G227–G237. doi:10.1152/ajpgi.00046.2020 32597706 PMC7500266

[11] Louie TJ , Cannon K , Byrne B , Emery J , Ward L , Eyben M , Krulicki W . 2012. Fidaxomicin preserves the intestinal microbiome during and after treatment of Clostridium difficile infection (CDI) and reduces both toxin reexpression and recurrence of CDI. Clin Infect Dis 55 Suppl 2:S132–42. doi:10.1093/cid/cis338 22752862 PMC3388020

[12] Mitra S , Chilton C , Freeman J , Wood H , Quirke P , Taylor M , Vickers R , Wilcox M . 2017. Preservation of gut microbiome following ridinilazole vs. fidaxomicin treatment of Clostridium difficile infection. Open Forum Infect Dis 4:S526–S527. doi:10.1093/ofid/ofx163.1372

[13] Snydman DR , McDermott LA , Jacobus NV , Thorpe C , Stone S , Jenkins SG , Goldstein EJC , Patel R , Forbes BA , Mirrett S , Johnson S , Gerding DN . 2015. U.S.-based national sentinel surveillance study for the epidemiology of Clostridium difficile-associated diarrheal isolates and their susceptibility to fidaxomicin. Antimicrob Agents Chemother 59:6437–6443. doi:10.1128/AAC.00845-15 26239985 PMC4576112

[14] Thorpe CM , McDermott LA , Tran MK , Chang J , Jenkins SG , Goldstein EJC , Patel R , Forbes BA , Johnson S , Gerding DN , Snydman DR . 2019. U.S.-based national surveillance for fidaxomicin susceptibility of Clostridioides difficile-associated diarrheal isolates from 2013 to 2016. Antimicrob Agents Chemother 63:e00391-19. doi:10.1128/AAC.00391-19 31085514 PMC6591623

[15] Rizzardi K , Åkerlund T , Norén T , Matussek A . 2020. Impact of ribotype on Clostridioides difficile diagnostics. Eur J Clin Microbiol Infect Dis 39:847–853. doi:10.1007/s10096-019-03772-z 31884555 PMC7182543

[16] Behroozian AA , Chludzinski JP , Lo ES , Ewing SA , Waslawski S , Newton DW , Young VB , Aronoff DM , Walk ST . 2013. Detection of mixed populations of Clostridium difficile from symptomatic patients using capillary-based polymerase chain reaction ribotyping. Infect Control Hosp Epidemiol 34:961–966. doi:10.1086/671728 23917911 PMC4016961

[17] Snydman DR , McDermott LA , Jenkins SG , Goldstein EJC , Patel R , Forbes BA , Johnson S , Gerding DN , Thorpe CM , Walk ST . 2020. Epidemiologic trends in C. difficile ribotypes in the United States, 2011-2016. Anaerobe 63:102185. doi:10.1016/j.anaerobe.2020.102185 32387171

[18] Johnson S , Lavergne V , Skinner AM , Gonzales-Luna AJ , Garey KW , Kelly CP , Wilcox MH . 2021. Clinical practice guideline by the infectious diseases society of America (IDSA) and society for Healthcare epidemiology of America (SHEA): 2021 focused update guidelines on management of Clostridioides difficile infection in adults. Clin Infect Dis 73:755–757. doi:10.1093/cid/ciab718 34492699

[19] Okhuysen PC , Ramesh M , Garey KW , Louie TJ , Cisneros JT , Stychneuskaya A , Kiknadze N , Li J , Duperchy E , Wilcox PMH , Montoya JG , Styles L , Clow F , James D , Dubberke ER , De Oliveira CM , Van Steenkiste C . 2022. Abstract presented at ID week. Ri-codify phase 3, randomized, double blind study to evaluate the efficacy and safety of ridinilazole compared to vancomycin for the treatment of Clostridioides difficile infection. Washington, DC.

[20] Knight DR , Kullin B , Androga GO , Barbut F , Eckert C , Johnson S , Spigaglia P , Tateda K , Tsai P-J , Riley TV . 2019. Evolutionary and genomic insights into Clostridioides difficile sequence type 11: a diverse zoonotic and antimicrobial-resistant lineage of global one health importance. mBio 10:e00446-19. doi:10.1128/mBio.00446-19 30992351 PMC6469969

[21] Moloney G , Eyre DW , Mac Aogáin M , McElroy MC , Vaughan A , Peto TEA , Crook DW , Rogers TR . 2021. Human and porcine transmission of Clostridioides difficile ribotype 078, Europe. Emerg Infect Dis 27:2294–2300. doi:10.3201/eid2709.203468 34423760 PMC8386809

[22] Álvarez-Pérez S , Blanco JL , Harmanus C , Kuijper E , García ME . 2017. Subyping and antimicrobial susceptibility of Clostridium difficile ribotype 078/126 isolates of human and animal origin. Vet Microbiol 199:15–22. doi:10.1016/j.vetmic.2016.12.001 28110780

[23] Guh AY , Mu Y , Winston LG , Johnston H , Olson D , Farley MM , Wilson LE , Holzbauer SM , Phipps EC , Dumyati GK , Beldavs ZG , Kainer MA , Karlsson M , Gerding DN , McDonald LC , Emerging Infections Program Clostridioides difficile Infection Working Group . 2020. Trends in U.S. burden of Clostridioides difficile infection and outcomes. N Engl J Med 382:1320–1330. doi:10.1056/NEJMoa1910215 32242357 PMC7861882

[24] George WL , Sutter VL , Citron D , Finegold SM . 1979. Selective and differential medium for isolation of Clostridium difficile. J Clin Microbiol 9:214–219. doi:10.1128/jcm.9.2.214-219.1979 429542 PMC272994

[25] Jousimies-Somer HR , Summanen P , Citron DM , Baron EJ , Wexler HA , Finegold SM . 2002. Wadsworth-KTL anaerobic Bacteriology manual. 6th ed. Star Publishing Company, Belmont, CA.

[26] Clinical and Laboratory Standards Institute . 2018. CLSI publication number M11-A9, In Methods for antimicrobial susceptibility testing of anaerobic bacteria. Clinical and Laboratory Standards Institute, Wayne, PA.

[27] Clinical and Laboratory Standards Institute . 2021. CLSI document M100-S31, In Performance standard for antimicrobial susceptibility testing. 31st Informational Supplement. Clinical and Laboratory Standards Institute, Wayne, PA.

[28] Tygacill . 2017. Pfizer product monograph. Pfizer, Kirkland, Canada. https://www.pfizer.ca/sites/default/files/201710/TYGACIL_PM.pdf.

[29] The European Committee on Antimicrobial Susceptibility Testing . 2021. Version. Breakpoint tables for interpretation of Mics and zone diameters, Vol. 11, p 0.

[30] Martinson JNV , Broadaway S , Lohman E , Johnson C , Alam MJ , Khaleduzzaman M , Garey KW , Schlackman J , Young VB , Santhosh K , Rao K , Lyons RH , Walk ST . 2015. Evaluation of portability and cost of a fluorescent PCR ribotyping protocol for Clostridium difficile epidemiology. J Clin Microbiol 53:1192–1197. doi:10.1128/JCM.03591-14 25631804 PMC4365229

